# Association Between the Degree of Extravalvular Cardiac Damage and the Severity of Aortic Stenosis

**DOI:** 10.1016/j.jacadv.2026.103227

**Published:** 2026-09-10

**Authors:** Augustin Coisne, Rubens Groulez, Camille Diharce, David Montaigne, Gilles Lemesle, Sandro Ninni, Samy Aghezzaf, Arnaud Sudre, Thomas Modine, André Vincentelli, Marie Jungling, Philippe Généreux, Sebastian Ludwig, Martin Leon, Juan Granada, Christophe Bauters

**Affiliations:** a Univ. Lille, Inserm, CHU Lille, Institut Pasteur de Lille, U1011-EGID, Lille, France; b Cardiovascular Research Foundation, New York City, New York, USA; c Heart and Lung Institute, University hospital of Lille, Lille, France; Univ. Lille, France; Institut Pasteur of Lille, Inserm U1011, Lille, France; FACT (French Alliance for Cardiovascular Trials), Paris, France; d Univ. Lille, Inserm, CHU Lille, Institut Pasteur de Lille, U1167, Lille, France; e UMCV, Hôpital Cardiologique Haut-Lévêque, CHU de Bordeaux, Pessac, France; f Gagnon Cardiovascular Institute, Morristown Medical Center, Morristown, New Jersey, USA; g Department of Cardiology, University Heart and Vascular Center Hamburg, University Medical Center Hamburg-Eppendorf, Hamburg, Germany

**Keywords:** aortic stenosis, aortic valve replacement, mortality

## Abstract

**Background:**

The relationship between the extent of extravalvular cardiac damage (CD) and valve disease severity remains uncertain in aortic stenosis (AS) patients.

**Objectives:**

The objective of the study was to investigate the correlation between AS severity and CD staging and its clinical impact across the spectrum of AS outpatients.

**Methods:**

Consecutive patients with mild (peak aortic velocity = 2.5-2.9 m/s), moderate (3-3.9 m/s), and severe AS (≥4 m/s) native AS from a regionwide cohort were included by 117 cardiologists between May 2016 and December 2017 in the VALVENOR cohort. Patients were followed up for 5 years.

**Results:**

Among the 1,747 patients with available CD (65% of the overall cohort), 744 (42.6%) patients had mild AS, 714 (40.9%) patients moderate AS, and 289 (16.5%) severe AS. Of the 1747 patients, 524 patients (30%) demonstrated CD stage 0, 298 (17%) stage 1, 765 (44%) stage 2, 109 (6%) stage 3, and 51 (3%) stage 4. Patients in stage >0 had higher peak aortic jet velocity compared to patients in stage 0 (3.35 ± 0.69 vs 3.11 ± 0.56 m/s; *P* < 0.001). After adjustment, higher peak aortic jet velocity (per m/s, *P* < 0.001) remained independently associated with CD stage >0. Both all-cause mortality and cardiovascular mortality were higher in patients in stage >0 as compared with patients in stage 0 (HR: 1.75; 95% CI: 1.44-2.11; *P* < 0.001 and HR: 2.52: 95% CI: 1.79-3.55; *P* < 0.001, respectively).

**Conclusions:**

The extent of extravalvular CD correlates with AS severity and is associated with poor clinical outcomes across all AS stages. Further research is warranted to determine whether assessing CD should be integrated in the management of patients with AS.

Aortic stenosis (AS) is the most frequent valvular heart disease in Western countries,^1^ affecting approximately 5% of individuals over 65 years.^2^ Traditionally, therapeutic decisions are mainly guided by the degree of valvular disease severity, that is, by transvalvular gradients and valve area measurements.^3^, ^4^, ^5^ However, there is increasing recognition that the burden of AS extends beyond the valve itself, involving a continuum of myocardial and extravalvular structural changes referred to as extravalvular cardiac damage (CD).^6^ These maladaptive changes are thought to result from chronic pressure overload, leading to a progressive cascade of left ventricular (LV) remodeling, diastolic dysfunction, left atrium (LA) dilatation, and ultimately systolic dysfunction.^7^ In this setting, a staging classification has been proposed to characterize the extent of these extravalvular CD including changes in the LV, LA, mitral valve, pulmonary vasculature, tricuspid valve, and right ventricle (RV).^8^

Although both surgical and transcatheter aortic valve replacement (AVR) can halt or partially reverse certain aspects of this CD, patients with more advanced stages of CD experience worse outcomes.^9^

Notably, most studies exploring the clinical implications of extravalvular CD in AS are generally restricted to patients with severe AS only or have been conducted in tertiary centers, including therefore a selected population. A better understanding of the prognosis of CD across the spectrum of AS could be essential for integrating a staging approach into clinical decision-making and refine risk stratification in AS. In addition, although the extent of extravalvular CD has been associated with quality of life^10^ and poor prognosis in asymptomatic patients with severe AS^11^ and in patients with moderate AS,^12^ the correlation between the severity of the valvular disease and the degree of CD is still debated.^13^

This study, based on a regionwide cohort of unselected outpatients, aimed at investigating the distribution and the association between AS severity and CD as well as exploring the clinical impact of extravalvular CD across the entire spectrum of AS, from mild to severe.

## Methods

The data that support the findings of this study are available from the corresponding author on reasonable request.

### Study population

The VALVENOR (Suivi d’une cohorte de patients présentant une sténose VALVulaire aortiquE en région NORd-pas-de-Calais) study enrolled 2,830 outpatients with native valvular AS between May 2016 and December 2017.^14^ Patients with a peak aortic jet velocity (Vmax) ≥2.5 m/s on transthoracic echocardiography (TTE) were prospectively included by 117 cardiologists from the Nord-Pas-de-Calais region in France during outpatient visits. Patients younger than 18 years or with a documented history of AVR were excluded. We also excluded 110 patients with concomitant severe aortic regurgitation, severe mitral stenosis or regurgitation, or prior mitral valve intervention, leaving 2,720 patients for the present analysis. Participating physicians were selected based on their geographic distribution to provide a representative sample of the current practice of all cardiology care in the region, including university public hospitals, nonuniversity public hospitals, and private practices. This study was approved by the French medical data protection committee (CCTIRS) and authorized by the Commission Nationale de l'Informatique et des Libertés for the treatment of personal health data. All patients consented to the study after being informed in writing of the study's objectives and treatment of the data, as well as on their rights to object, of access, and of rectification.

### Transthoracic echocardiography

TTE was performed as part of routine clinical practice using commercially available systems. Vmax was derived from transaortic flow, recorded with continuous wave Doppler. According to current diagnostic criteria^15^ and as previously described,^14^^,^^16^^,^^17^ patients were categorized as follows: mild AS (Vmax 2.5-2.9 m/s), moderate AS (Vmax 3-3.9 m/s), and severe AS (Vmax ≥4 m/s).

### Assessment of cardiac damage

Extravalvular CD was assessed on echocardiography according to the classification proposed by Généreux et al.^8^ Patients were classified into the following 5 stages: stage 0, no extravalvular CD; stage 1, LV damage (systolic LV dysfunction, LV hypertrophy, and LV diastolic dysfunction grade >II); stage 2, LA or mitral valve damage (LA dilatation, ≥ moderate mitral regurgitation, or presence of atrial fibrillation); stage 3, pulmonary vasculature or tricuspid valve damage (pulmonary hypertension, ≥ moderate tricuspid regurgitation); and stage 4, RV damage (systolic RV dysfunction). RV dysfunction was assessed on the basis of quantitative criteria, following the guideline recommendations. Patients were hierarchically classified into the most advanced CD stage if at least 1 of the criteria was met within that stage. Missing variables were not assumed to indicate the absence of CD. Conversely, patients were classified into a given CD stage whenever at least 1 criterion for that stage was present, even if data required to assess lower stages were unavailable.

### Follow-up

Patients were followed up by their treating cardiologists. The number of outpatient visits during follow-up was at the discretion of the cardiologists. The individual decision to perform or contraindicate patients for AVR was left to treating cardiologists and local Heart Teams. Protocol-specified follow-up was performed at 2 years and 5 years using standardized case record forms to report clinical events and cardiovascular procedures. To minimize follow-up bias, general practitioners and/or patients were contacted by a research technician in the case of missing information. Five-year follow-up was completed in 2,704 patients (99.4%). All clinical events and cardiovascular procedures were adjudicated by 2 investigators blinded to each other (A.C., D.M., S.A., or C.B.). A third investigator (A.C., D.M., S.A., or C.B.) joined the adjudication in case of disagreement according to prespecified definitions. A consensus was then reached.

Cardiovascular causes of death included congestive heart failure (HF), sudden death, stroke, limb ischemia, myocardial infarction, and other cardiovascular death. Noncardiovascular causes of death included cancer, sepsis, renal failure, respiratory failure, suicide or accident, and other noncardiovascular death. Deaths by an unknown cause were kept as a separate category. The definitions for adjudication of the causes of death were published previously.^18^

### Statistical analysis

Statistical analyses were performed with the STATA 14.2 software (StataCorp). Continuous variables were described as mean ± SD. Categorical variables were presented as absolute numbers and percentages. Baseline characteristics were compared using analysis of variance or the Student’s unpaired *t* test for continuous variables and the chi-square or the Fisher exact test for categorical variables. Factors associated with CD stage >0 were assessed by logistic regression; ORs and 95% CIs are shown. Mortality rates were analyzed by censoring data at time of AVR. All-cause mortality was estimated using the Kaplan-Meier method. For cardiovascular mortality, cumulative incidence functions are shown with death from any other cause as the competing event. HRs and 95% CIs were calculated with the Cox model.

## Results

### Study population

**Figure 1:**
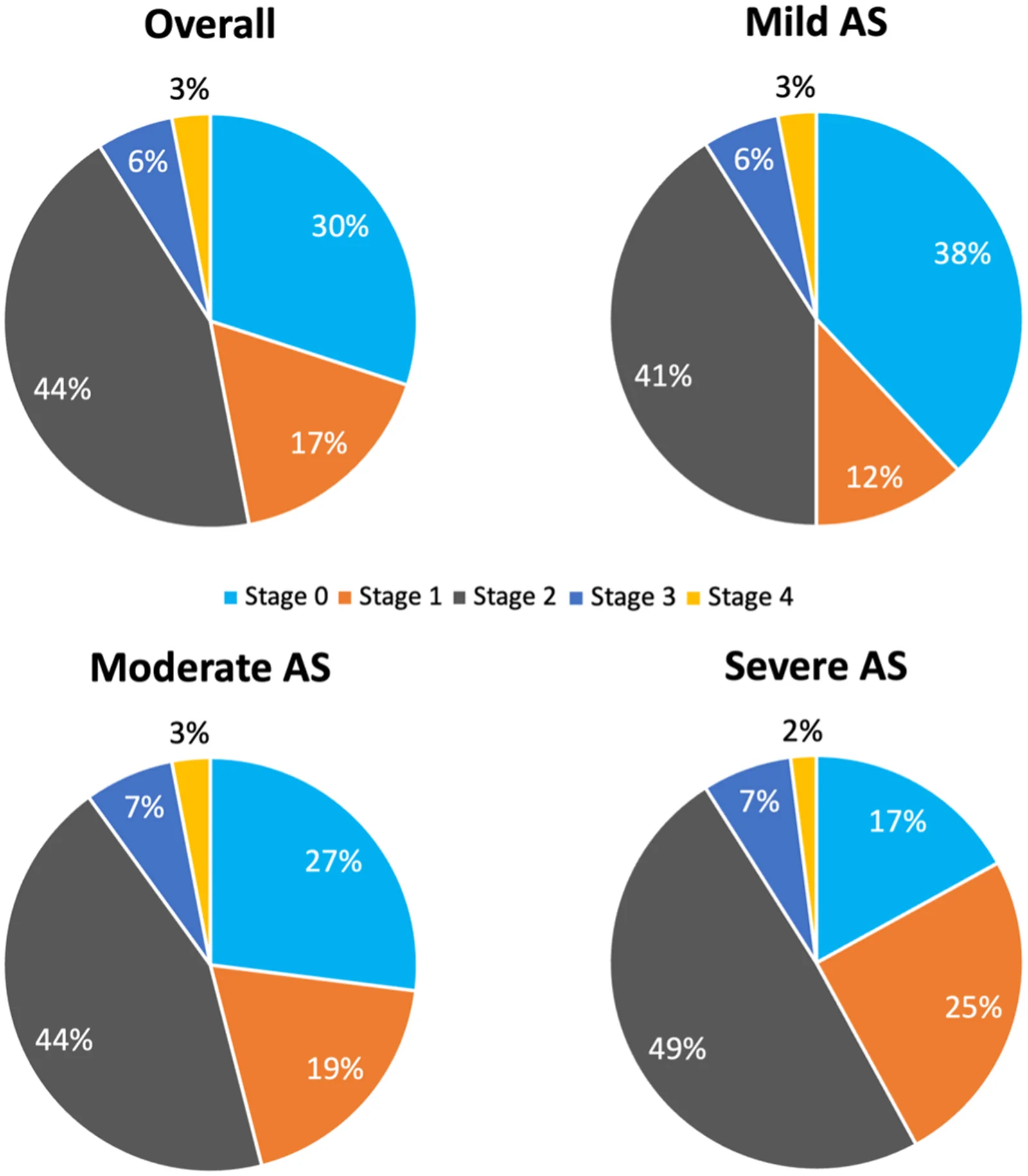
Distribution of Cardiac Damage Stage According to AS Severity Among the 1747 patients from the Valvenor cohort, 30% demonstrated CD stage 0, 17% stage 1, 44% stage 2, 6% stage 3 and 3% stage 4. AS = aortic stenosis.

Among the 2,704 patients included in the VALVENOR registry, extravalvular CD was evaluable in 1747 patients (65%). There was no significant difference between patients with no staging available and those with staging available except for previous hospitalizations for HF (Supplemental Table 1). A total of 744 patients (42.6%) had mild AS, 714 (40.9%) had moderate AS, and 289 (16.5%) had severe AS. Of the 1747 patients, 524 patients (30%) demonstrated CD stage 0, 298 (17%) stage 1, 765 (44%) stage 2, 109 (6%) stage 3, and 51 (3%) stage 4 (Figure 1). AVR rates according to extravalvular CD stage are presented in Supplemental Table 2.

### Correlates of CD

Advancing CD stage was associated with higher prevalence of comorbidities including prior coronary event, hypertension, diabetes mellitus, and prior HF hospitalization (Table 1). No significant differences in sex distribution were observed across CD stages. Similar findings were observed when the analysis was restricted to patients with mild AS (Supplemental Table 3), moderate AS (Supplemental Table 4), or severe AS (Supplemental Table 5).

**Table 1:** Baseline Characteristics of the Study Population According to Cardiac Damage Staging

	Stage 0 (n = 524)	Stage 1 (n = 298)	Stage 2 (n = 765)	Stage 3 (n = 109)	Stage 4 (n = 51)	*P* Value
Age (y)	73.1 ± 12.4	76.1 ± 10.3	77.4 ± 10.4	79.3 ± 10.3	76 ± 9.9	<0.001
Women	239 (45.6)	138 (46.3)	364 (47.6)	65 (59.6)	23 (45.1)	0.113
History of hypertension	375 (71.6)	226 (75.8)	614 (80.3)	74 (67.9)	39 (76.5)	0.002
Diabetes mellitus	149 (28.4)	90 (30.2)	237 (31)	21 (19.3)	22 (43.1)	0.025
Previous coronary event	78 (14.9)	58 (19.5)	150 (19.6)	22 (20.2)	23 (45.1)	<0.001
Previous myocardial infarction	39 (7.4)	29 (9.7)	77 (10.1)	13 (11.9)	15 (29.4)	<0.001
Previous coronary bypass	13 (2.5)	15 (5)	37 (4.8)	9 (8.3)	12 (23.5)	<0.001
Previous PCI	58 (11.1)	42 (14.1)	107 (14)	14 (12.8)	8 (15.7)	0.559
Previous hospitalization for HF	18 (3.4)	30 (10.1)	104 (13.6)	26 (23.9)	21 (41.2)	<0.001
Previous stroke	36 (6.9)	17 (5.7)	87 (11.4)	4 (3.7)	6 (11.8)	0.002
NYHA functional class at inclusion^a^						<0.001
I	252 (48.3)	104 (35.1)	272 (35.9)	25 (23.1)	6 (12.2)	
II	227 (43.5)	157 (53.1)	391 (51.6)	54 (50)	21 (42.9)	
III-IV	43 (8.2)	35 (11.8)	95 (12.5)	29 (26.9)	22 (44.9)	
Angina at inclusion	16 (3.1)	19 (6.4)	35 (4.6)	6 (5.5)	1 (2)	0.191
Beta-blocker	192 (36.6)	129 (43.3)	398 (52)	58 (53.2)	32 (62.8)	<0.001
ACEI or ARB	325 (62)	193 (64.8)	523 (68.4)	75 (68.8)	36 (70.6)	0.155
Statin	276 (52.7)	151 (50.7)	448 (58.6)	59 (54.1)	29 (56.9)	0.115
Antiplatelet	254 (48.5)	153 (51.3)	338 (44.2)	39 (35.8)	21 (41.2)	0.029
Oral anticoagulant	65 (12.4)	44 (14.8)	243 (31.8)	35 (32.1)	26 (51)	<0.001

Values are mean ± SD or n (%).

*P* value by ANOVA or chi-square.

ACEI = angiotensin-converting enzyme inhibitor; ARB = angiotensin 2 receptor antagonist; HF = heart failure; PCI = percutaneous coronary intervention.

a Missing data in 14 patients.

Importantly, the prevalence of CD stage 0 decreased as AS severity increased, observed in 38%, 27%, and 17% of patients with mild, moderate, and severe AS, respectively (*P* < 0.001) (Figure 1). Conversely, the prevalence of CD stages 1 and 2 increased progressively with AS severity: stage 1 was present in 12%, 19%, and 25%, whereas stage 2 was found in 41%, 44%, and 49% of patients with mild, moderate, and severe AS, respectively.

**Figure 2:**
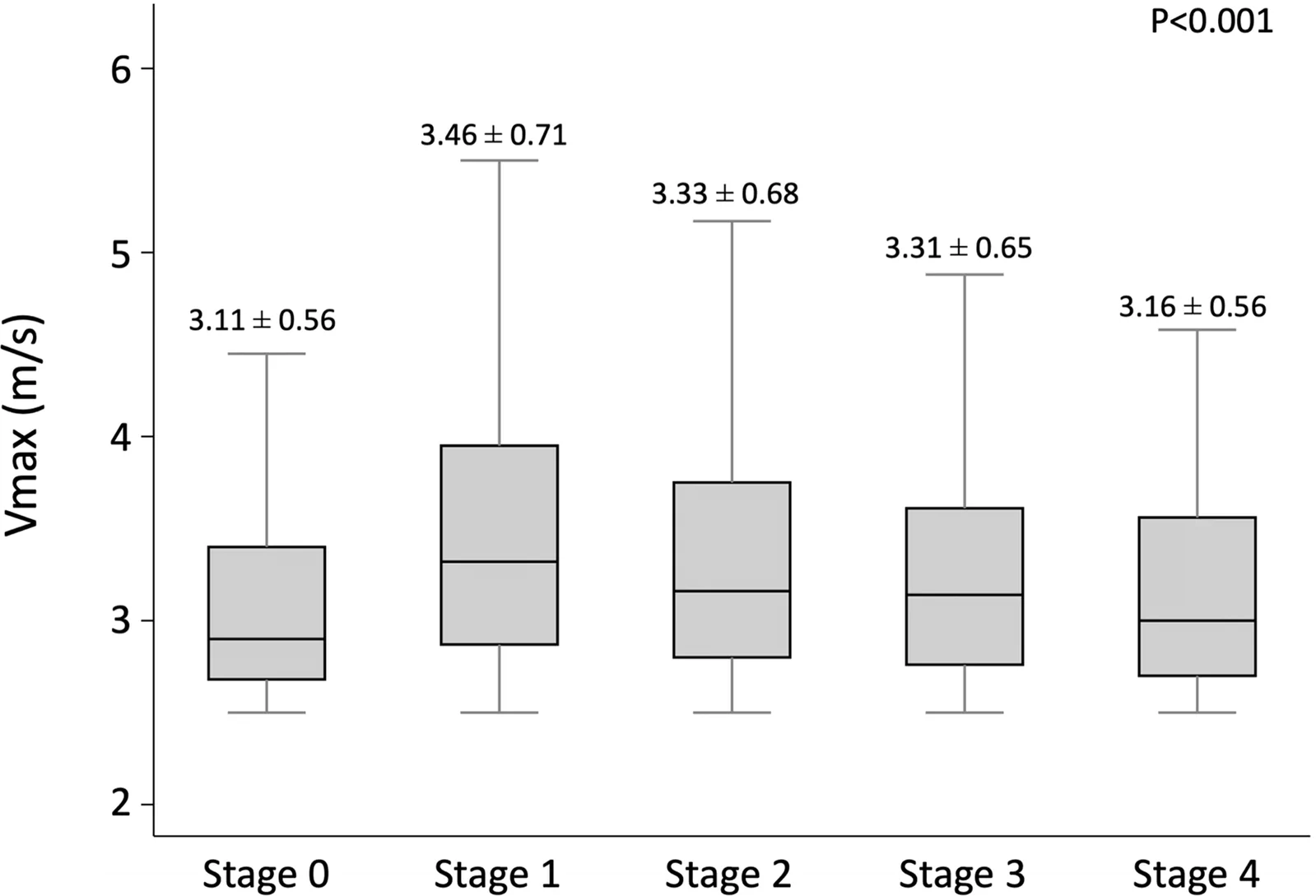
Vmax According to Cardiac Damage Stage Patients in stage >0 had higher Vmax compared to patients in stage 0 (3.35 ± 0.69 vs 3.11 ± 0.56 m/s, *P* < 0.001). Values reported above each graph correspond to the mean ± SD. Box-and-whisker plots display the median, the 25th and 75th percentiles, and the adjacent values. Vmax = peak aortic jet velocity.

Table 2 presents the echocardiographic characteristics of the study population according to CD staging in the VALVENOR cohort. Figure 2 illustrates the Vmax values for each of the 5 CD stages. Patients in stage >0 had higher Vmax compared to patients in stage 0 (3.35 ± 0.69 vs 3.11 ± 0.56 m/s; *P* < 0.001).

**Table 2:** Echocardiographic Characteristics of the Study Population According to Cardiac Damage Staging

	Stage 0 (n = 524)	Stage 1 (n = 298)	Stage 2 (n = 765)	Stage 3 (n = 109)	Stage 4 (n = 51)	*P* Value
Mild AS, n (%)	283 (54)	89 (29.9)	305 (39.9)	43 (39.5)	24 (47)	
Moderate AS, n (%)	190 (36.3)	137 (46)	319 (41.7)	47 (43.1)	21 (41.2)	<0.001
Severe AS, n (%)	51 (9.7)	72 (24.1)	141 (18.4)	19 (17.4)	6 (11.8)	
LVEF^a^, %	66 ± 7	64 ± 10	64 ± 10	64 ± 11	54 ± 15	<0.001
LVEF <50%^b^, n (%)	0	27 (9.3)	48 (6.3)	7 (6.4)	15 (30)	<0.001
LV hypertrophy^c^, n (%)	0	270 (93.8)	361 (55)	41 (48.2)	18 (46.2)	<0.001
LAVi >34 mL/m^2,^^d^, n (%)	0	0	732 (97.6)	58 (71.6)	36 (78.3)	<0.001
Moderate MR^e^, n (%)	0	0	130 (19.9)	26 (27.1)	6 (13.3)	<0.001
SPAP ≥60 mm Hg^f^, n (%)	0	0	0	37 (35.6)	8 (16.7)	<0.001
≥ moderate TR^g^, n (%)	0	0	0	76 (83.5)	8 (21.1)	<0.001
RV dysfunction, n (%)	0	0	0	0	51 (100)	<0.001

Values are mean ± SD or n (%).

*P* value by ANOVA or chi-square.

AS = aortic stenosis; LAVi = left atrium volume index; LV = left ventricle; LVEF = left ventricular ejection fraction; MR = mitral regurgitation; RV = right ventricle; SPAP = systolic pulmonary artery pressure; TR = tricuspid regurgitation.

a Missing data in 252 patients.

b Missing data in 23 patients.

c Missing data in 317 patients.

d Missing data in 365 patients.

e Missing data in 336 patients.

f Missing data in 21 patients.

g Missing data in 534 patients.

In multivariable analysis, older age (per year, *P* < 0.001), prior coronary event (*P* = 0.04), prior hospitalization for HF (*P* < 0.001), NYHA functional class >I (*P* = 0.03) and higher Vmax (per m/s, *P* < 0.001) were all independently associated with CD stage >0 (Table 3). Similar findings were observed when AS severity categories were analyzed using mild AS as the reference group (Supplemental Table 6).

**Table 3:** Factors Associated With CD Stage >0 (Logistic Regression)

	Univariable		Multivariable	
	OR (95% CI)	*P* Value	OR (95% CI)	*P* Value
Age, per year	1.03 (1.02-1.04)	<0.001	1.03 (1.02-1.04)	**<0.001**
Women	1.11 (0.91-1.36)	0.313	0.95 (0.75-1.19)	0.644
History of hypertension	1.40 (1.11-1.77)	0.004	1.27 (0.98-1.64)	0.069
Diabetes mellitus	1.09 (0.87-1.37)	0.446	1.06 (0.84-1.36)	0.612
Previous coronary event	1.49 (1.13-1.97)	0.005	1.37 (1.01-1.84)	**0.041**
Previous hospitalization for HF	4.88 (2.97-8.02)	<0.001	3.98 (2.39-6.61)	**<0.001**
Previous stroke	1.39 (0.94-2.06)	0.095	1.20 (0.79-1.80)	0.394
NYHA functional class >I	1.84 (1.50-2.28)	<0.001	1.29 (1.02-1.62)	**0.031**
Angina at inclusion	1.67 (0.95-2.92)	0.074	1.25 (0.69-2.25)	0.457
Vmax, per m/s	1.89 (1.59-2.27)	<0.001	1.92 (1.60-2.31)	**<0.001**

**Bold** values indicate at *P* < 0.05.

CD = cardiac damage; Vmax = peak aortic jet velocity; other abbreviation as in Table 1.

### Clinical outcomes

**Figure 3:**
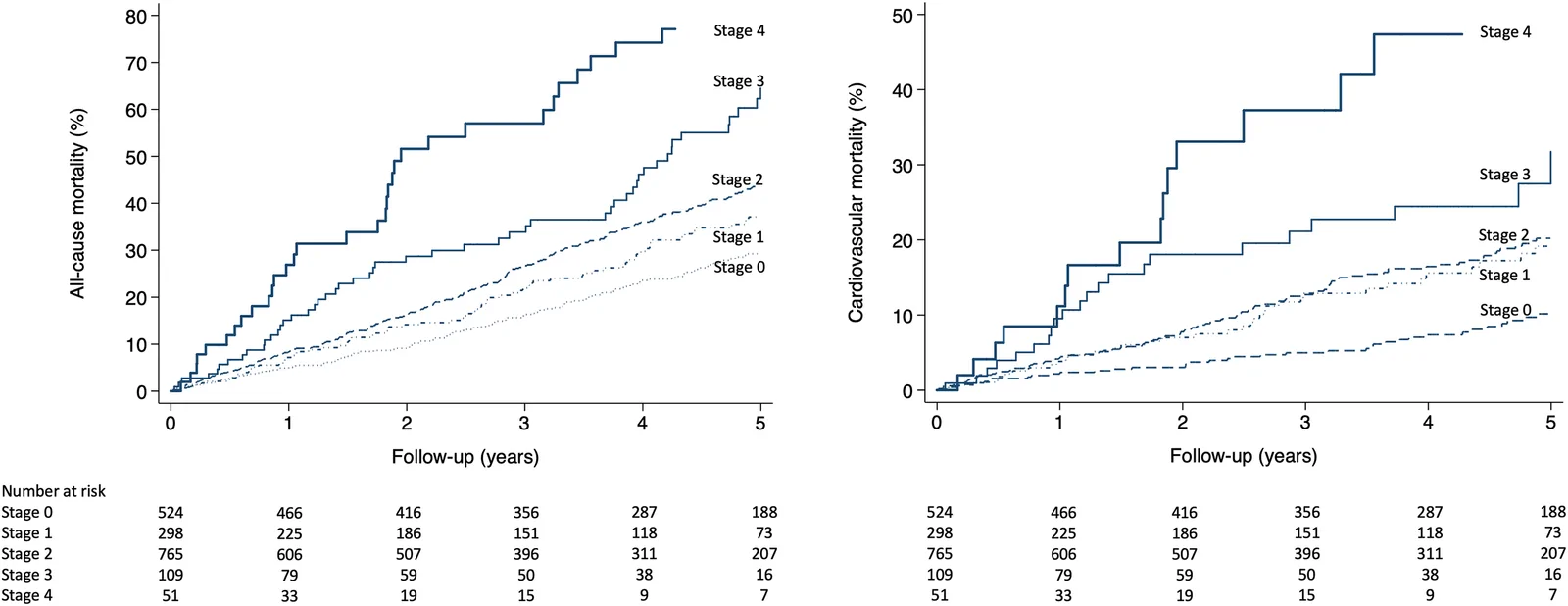
Outcomes According to Cardiac Damage Stages Cumulative risk of all-cause mortality (left panel) and cardiovascular mortality (right panel) during follow-up stratified by CD stage.

The 5-year all-cause and cardiovascular mortality rates were 30% and 10% in patients with CD stage 0, 34% and 18% in stage 1, 41% and 19% in stage 2, 58% and 30% in stage 3, and 71% and 41% in stage 4, respectively (Figure 3).

**Figure 4:**
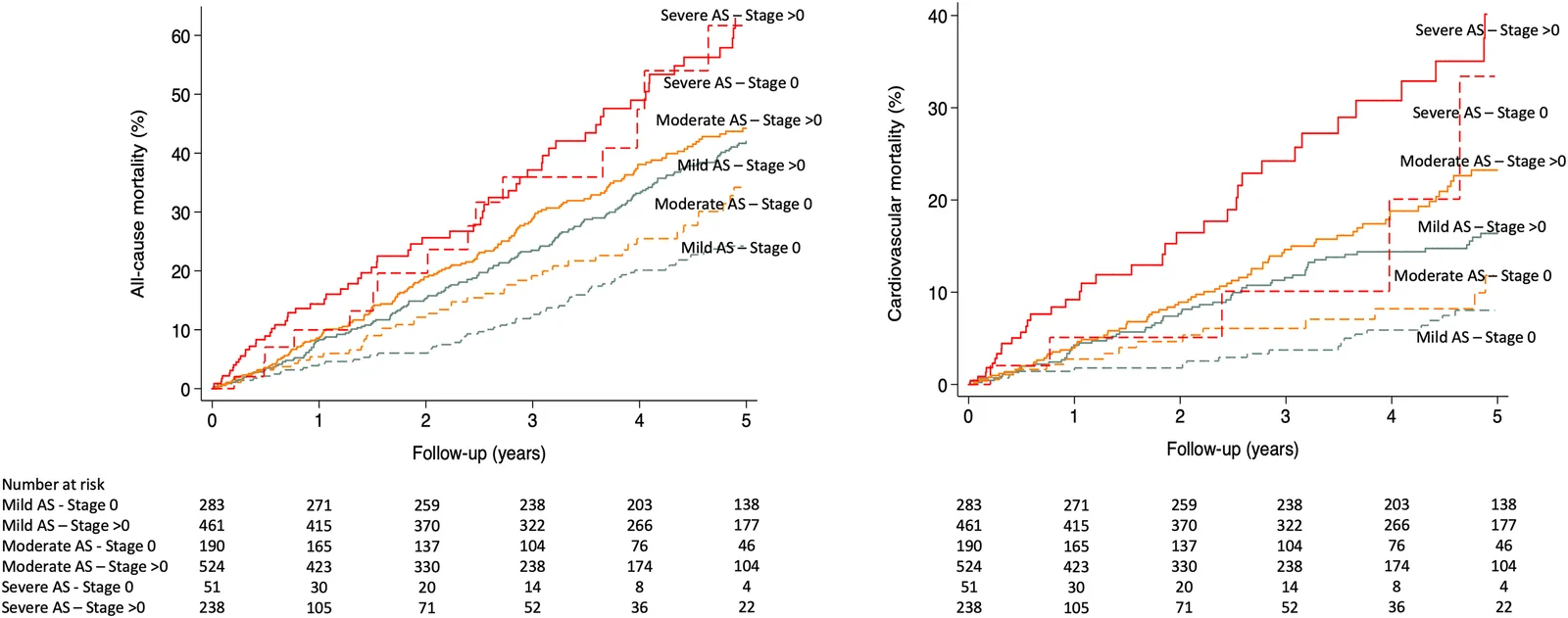
Outcomes According to Cardiac Damage Stages and AS Severity Cumulative risk of all-cause mortality (left panel) and cardiovascular mortality (right panel) during follow-up stratified by CD stage and AS severity. Abbreviation as in Figure 1.

Patients with CD stage >0 had higher all-cause mortality compared with patients in CD stage 0 in the overall cohort (HR: 1.75; 95% CI: 1.44-2.11; *P* < 0.001), in the mild AS group (HR: 1.84; 95% CI: 1.41-2.39; *P* < 0.001), in the moderate AS group (HR: 1.54; 95% CI: 1.12-2.12; *P* = 0.007), but not in the severe AS group (HR: 1.17; 95% CI: 0.67-2.05; *P* = 0.582) (Figure 4, Table 4). Similar findings were observed for cardiovascular mortality in the overall cohort (HR: 2.52; 95% CI: 1.79-3.55; *P* < 0.001), in the mild AS group (HR: 2.29; 95% CI: 1.40-3.73; *P* < 0.001), in the moderate AS group (HR: 2.34; 95% CI: 1.33-4.11; *P* = 0.003), and in the severe AS group (HR: 1.97; 95% CI: 0.77-5.00; *P* = 0.155). There was no evidence for a differential effect of CD across subgroups of AS severity (interaction *P* = 0.234 for all-cause and 0.990 for cardiovascular mortality).

**Table 4:** Prognostic Impact of CD Stage >0 (Versus CD Stage 0) in the Study Population and Stratified by AS Severity at Inclusion

	HR (95% CI)	*P* Value	Interaction *P* Value
All-cause mortality			
All patients (n = 1747)	1.75 (1.44-2.11)	<0.001	
Mild AS (n = 744)	1.84 (1.41-2.39)	<0.001	
Moderate AS (n = 714)	1.54 (1.12-2.12)	0.007	0.234
Severe AS (n = 289)	1.17 (0.67-2.05)	0.582	
Cardiovascular mortality			
All patients (n = 1747)	2.52 (1.79-3.55)	<0.001	
Mild AS (n = 744)	2.29 (1.40-3.73)	0.001	
Moderate AS (n = 714)	2.34 (1.33-4.14)	0.003	0.990
Severe AS (n = 289)	1.97 (0.77-5.00)	0.155	

Interaction *P* values correspond to tests for trend.

Abbreviations as in Tables 2 and 3.

Cox analysis using CD stage as separate categories, with stage 0 as the reference, is provided in Supplemental Table 7. Finally, the prognostic impact of CD stage >0 vs CD stage 0 remained significant in sensitivity analyzes without censoring at the time of AVR and with adjustment for AVR included as a time-dependent covariate (Supplemental Tables 8 and 9).

## Discussion

Exploring 1,747 outpatients from a regionwide cohort, we observed the following (Central Illustration): 1) the prevalence of CD stage 0 declined and stages 1 to 2 increased with AS hemodynamic severity; 2) higher Vmax was independently associated with CD stage >0; 3) CD stage >0 was associated with higher all-cause and cardiovascular mortality; and 4) there was no evidence for a differential effect on outcomes of CD across subgroups of AS severity.

**Central Illustration:**
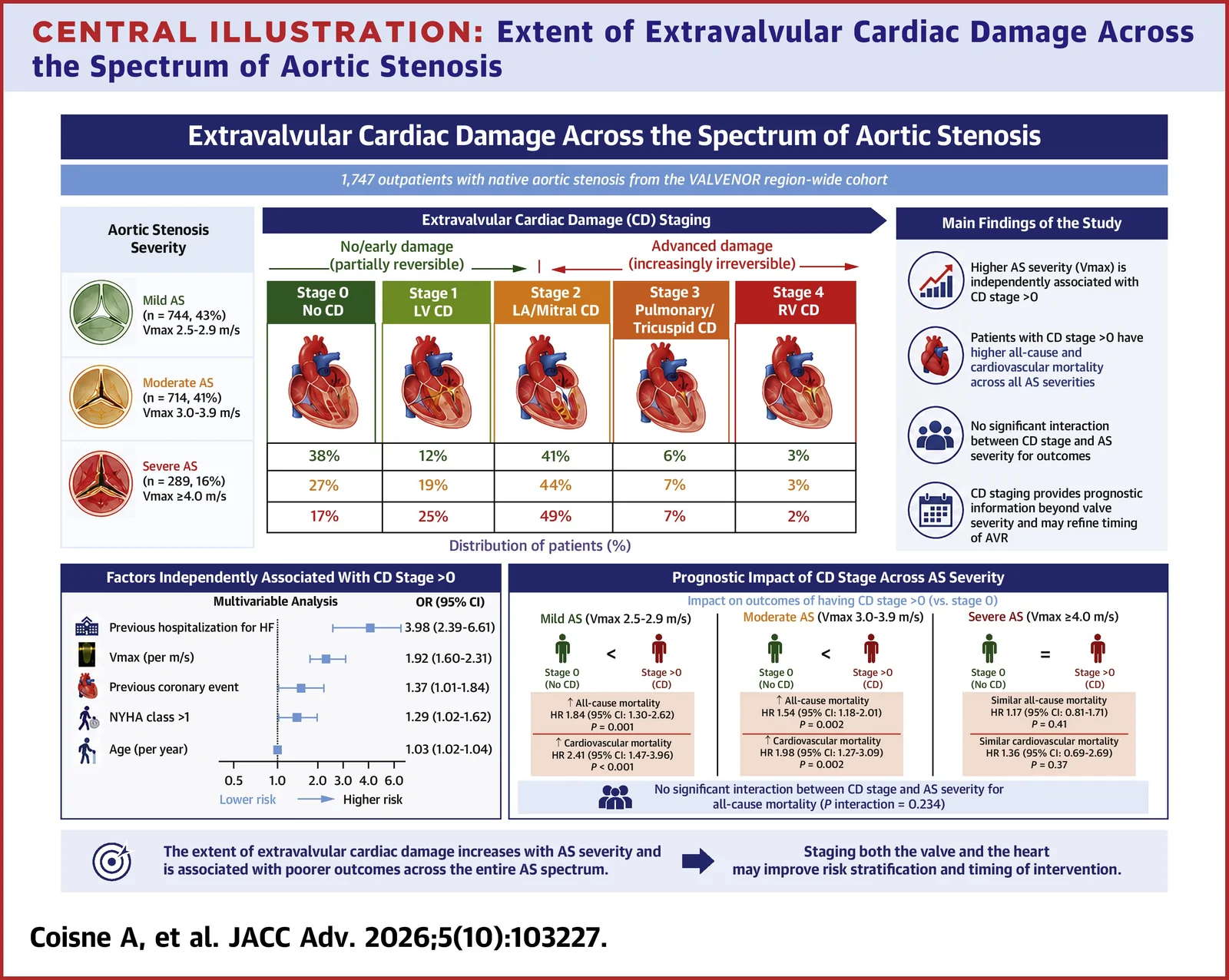
Extent of Extravalvular Cardiac Damage Across the Spectrum of Aortic Stenosis AS = aortic stenosis; AVR = aortic valve replacement; HF = heart failure; LA = left atrium; LV = left ventricle; RV = right ventricle; Vmax = peak aortic jet velocity.

### Prevalence and distribution

To provide a comprehensive perspective, we summarize the distribution of disease stages reported in several studies in Supplemental Table 10.

Multiple studies across varying AS severity grades consistently show that most patients present with stage 2 extravalvular CD. For example, in the PARTNER 2 trials, Généreux et al^8^ reported that ∼3% of patients were in stage 1, 50% in stage 2, and more than 70% in stage ≥2 at the time of transcatheter AVR. In contrast, Amanulla et al.^12^, analyzing 1,245 patients with moderate AS, found a higher proportion of patients in stage 1 (13.1%) and lower proportions in stage 2 (42.6%) and ≥2 (60.1%). Among patients with mild AS, Dahl et al^13^ observed that 17% were in stage 1, 50% in stage 2, and 63% in stage ≥2. Notably, substantial variability exists across studies—even when restricted to severe AS cohorts. Reported distributions range widely, with stage 0 from 1% to 30%, stage 2 from 9% to 62%, and stage 4 from 1.2% to 62%. Moreover, in patients with severe AS, the distribution of extravalvular CD also depends on flow status: patients with low-flow low-gradient AS (classical or paradoxical) showed higher proportions of stage 3 and 4.^19^

Recently, Angellotti et al^20^ examined 343 patients with moderate AS and 282 with severe AS enrolled over a 14-year period (2009-2023) in 2 centers and reported a similar distribution of CD stages between patients with moderate AS and those with severe AS. In contrast, in our regionwide outpatient cohort encompassing the full spectrum of AS severity, we observed a marked gradient in extravalvular CD distribution: the prevalence of CD stage 0 progressively declined with increasing AS severity, whereas stages 1 and 2 became more frequent. These discrepancies likely reflect differences in study period, sample size—both overall and within each AS severity stratum—and patient recruitment pathways. Importantly, stage distribution is expected to differ between community-based and tertiary-center cohorts. Community cohorts include more mild and moderate AS, capture earlier phases of cardiac remodeling, and therefore contain a greater proportion of stage 0 to 2 patients. Conversely, tertiary centers—affected by referral bias—tend to include more advanced disease, with higher proportions of stages 2 to 4.

Together, these observations highlight that CD staging is influenced not only by the degree of valvular stenosis but also by the clinical setting in which patients are assessed, underscoring the need for early referral before progression to advanced CD stages.

### Association between extravalvular CD and AS severity

Although extravalvular CD can theoretically be attributed to pathophysiologic remodeling from chronic pressure overload, its extent in AS is not determined solely by valve severity. Dahl et al^13^ demonstrated a strong association between CD and comorbidities, with the relationship to valve hemodynamics disappearing after adjustment. In contrast, we found that the presence of extravalvular CD (stage >0) was independently associated not only with age, prior coronary events, HF hospitalization, and higher NYHA functional class, but also with higher Vmax. These differences likely reflect variation in study populations, with different AS severity and comorbidity profiles.

Overall, CD likely reflects the combined effects of valvular obstruction and the underlying comorbidity burden rather than valve severity alone. Accordingly, advanced CD observed in patients with moderate AS may partly represent overlapping HF with preserved ejection fraction physiology, particularly in older patients with hypertension, atrial fibrillation, and diastolic dysfunction. Consistent with this interpretation, Figure 2 demonstrates substantial overlap in Vmax distributions across CD stages.

Although cardiac involvement cannot be explained exclusively by valve disease, integrating both valvular severity and cardiac impact may provide a more complete framework for patient management.^6^

It is also important to recognize that the current CD staging classification could be further refined by incorporating additional markers of myocardial and valvular disease to improve risk stratification, such as myocardial deformation imaging, cardiac magnetic resonance fibrosis assessment, or computed tomography–based valvular calcium quantification. In particular, a staging system incorporating multichamber myocardial deformation by TTE myocardial deformation assessed by speckle-tracking echocardiography has been shown to be more strongly associated with clinical outcomes than previously validated staging system.^21^

### Prognostic impact of extravalvular CD

Although both surgical and transcatheter AVR can halt or partially reverse aspects of CD, the extravalvular CD staging system has consistently shown incremental prognostic value for both short- and long-term outcomes after AVR.^9^ The prognostic impact of this system was first described in the PARTNER 2 trial, which enrolled intermediate-risk patients with severe symptomatic AS undergoing AVR^8^ and was subsequently validated in registries from 2 academic centers.^22^ More recently, a pooled meta-analysis of nearly 15,000 patients confirmed that each incremental stage of damage was associated with a stepwise increase in all-cause and cardiovascular mortality, independent of conventional surgical risk scores.^23^

Importantly, the prognostic implications extend beyond the perioperative period. Longitudinal studies demonstrated that baseline CD stage predicted persistent symptoms, impaired quality of life, increased costs, and greater health care utilization after AVR.^10^^,^^24^ Notably, patients with stage 3 or 4 damage often fail to experience reverse remodeling, underscoring the largely irreversible nature of advanced myocardial and right-sided involvement.^9^^,^^25^^,^^26^ This observation likely reflects the transition from initially adaptive remodeling in response to chronic pressure overload toward irreversible myocardial fibrosis and right-sided dysfunction, beyond which reverse remodeling following AVR becomes less likely. These findings support the concept that CD staging may help identify patients approaching this critical transition. Notably, the prognostic impact of CD staging has since been confirmed in severe asymptomatic AS,^11^ moderate AS,^12^ and even in mild AS patients.^13^

In our regionwide outpatient cohort, patients with CD stage >0 had higher all-cause and cardiovascular mortality compared with stage 0 with no evidence for a differential effect of CD across subgroups of AS severity. Notably, no significant prognostic impact of CD stage >0 was observed among patients with severe AS. This finding may be attributable to several factors, including the competing effect of AVR, referral bias and the relatively small sample size of this subgroup. In addition, the low prevalence of stage 0 among patients with severe AS may have limited the discriminatory capacity of the staging classification within this subgroup.

### Therapeutic implications

Recognition of the prognostic weight of extravalvular CD has major clinical implications. First, staging provides a simple, reproducible framework for risk stratification across both surgical AVR and transcatheter AVR candidates. Integration of this staging into multidisciplinary decision-making can help align therapeutic choices with patient prognosis. Moreover, incorporation into predictive models may improve patient counseling and trial design.^27^ Second, it underscores the limitations of relying solely on valve hemodynamics for timing AVR. Current guidelines emphasize symptoms and LV ejection fraction, but data suggest that once patients progress to stage 3 or 4, outcomes remain poor even after successful valve replacement. Therefore, earlier valve intervention, before the development of irreversible myocardial and right-sided involvement, may improve outcomes in selected patients, particularly those with moderate AS and evidence of myocardial decompensation. However, this hypothesis remains unproven and is currently being evaluated in ongoing randomized trials such as PROGRESS and EXPAND II. Any potential benefit must be balanced against procedural risks, long-term valve durability, and the potential role of more durable surgical options, such as the Ross procedure, in selected younger patients.

Finally, the concept of CD beyond the valve highlights the potential role of adjunctive therapies targeting myocardial fibrosis, atrial remodeling, or pulmonary hypertension. Although AVR addresses the valvular obstruction, tailored pharmacologic or device-based therapies may mitigate residual risk, particularly in advanced stages.

Collectively, these findings support a paradigm in which both the severity of valvular obstruction and the extent of myocardial damage should be considered to more accurately define clinically significant AS.^28^ Because some components of CD may be driven by comorbid conditions that are unlikely to improve following valve intervention alone, comprehensive cardiovascular optimization should be considered before, or in conjunction with, early aortic valve intervention.

### Limitations and future directions

Despite robust evidence, several limitations should be acknowledged. First, this observational study cannot establish a causal relationship between AS severity and the extent of extravalvular CD. Patients with more advanced CD also exhibited a greater burden of cardiovascular comorbidities, which may have contributed to both cardiac remodeling and adverse outcomes despite multivariable adjustment. Second, CD staging was available in only 65% of the overall VALVENOR cohort, potentially limiting the generalizability of our findings, although patients with and without available staging had largely similar baseline characteristics. Third, the staging system, although pragmatic, is semiquantitative and relies largely on echocardiographic surrogates. To provide a clinically relevant and readily applicable message while reflecting current cardiology practice, we categorized AS severity according to Vmax alone. Although Vmax may underestimate AS severity in patients with low-flow states, its robust prognostic value has been consistently demonstrated in several studies.^29^ Nevertheless, we acknowledge that low-flow states may have partly mediated the observed association between AS severity and CD. Fourth, serial echocardiographic assessment of CD staging after AVR were not systematically collected within the VALVENOR registry precluding evaluation of the temporal evolution and reversibility of CD after AVR within the VALVENOR registry. Fifth, for prognostic analyses, CD was dichotomized (stage 0 vs stage >0), which simplified interpretation but reduced the granularity of the original five-stage classification and may have attenuated stage-specific prognostic differences. Finally, most available data derive from post hoc analyses or observational cohorts; randomized trials addressing intervention timing based on extravalvular stage are lacking. Ongoing studies will clarify whether early AVR in asymptomatic patients with subclinical extravalvular CD improves outcomes.

## Conclusions

In a regionwide cohort, we found that the extent of extravalvular CD correlates with AS severity and is associated with poor outcomes across all stages. Further research is warranted to determine whether assessing CD should be integrated in the management of patients with AS.

Perspectives **COMPETENCY IN MEDICAL KNOWLEDGE:** Our findings demonstrate that the degree of extravalvular CD correlates with the severity of AS and is associated with adverse outcomes at every disease stage.

**TRANSLATIONAL OUTLOOK:** Further studies are needed to determine whether evaluating CD should become part of routine management for patients with AS, and whether early AVR in asymptomatic individuals with subclinical extravalvular CD could improve outcomes.

## Funding support and author disclosures

This study was supported by a grant from Fédération Française de Cardiologie. Dr Montaigne is supported by grants from Agence Nationale pour la Recherche (ANR-10-LABX-0046, ANR TOMIS-Leukocyte: ANR-CE14-0003-01 and ANR CALMOS: ANR-18-CE17-0003-02), the Leducq Foundation LEAN Network 16CVD01 and the National Center for Precision Diabetic Medicine – PreciDIAB (ANR-18-IBHU-0001; 20001891/NP0025517; 2019_ESR_11). Dr Coisne is proctor for Abbott Vascular and received speaker fees for Abbott Vascular, Bristol Myers Squibb, Edwards Lifescience, GE Healthcare, MSD, and Pfizer. Dr Lemesle has received honoraria and fees from Alnylam, Amarin, Amgen, AstraZeneca, Bayer, Bristol Myers Squibb, Boehringer Ingelheim, MSD, Novartis, Novonordisk, Organon, Pfizer, and Sanofi Aventis. Dr Modine is consultant for Abbott Medical, Edwards Lifescience, and Medtronic; received grants from Abbott Medical, Edwards Lifescience, and Medtronic; and serves in the scientific advisory board of Medtronic. Dr Leon has received institutional research grants from Abbott, Boston Scientific, and Medtronic; has been an unpaid advisor for Abbott, Boston Scientific, Sinomed, and Medtronic; and holds equity in Medinol. All other authors have reported that they have no relationships relevant to the contents of this paper to disclose.

## References

[1] Nkomo V.T., Gardin J.M., Skelton T.N., Gottdiener J.S., Scott C.G., Enriquez-Sarano M. (2006). Burden of valvular heart diseases: a population-based study. Lancet Lond Engl.

[2] Benjamin E.J., Virani S.S., Callaway C.W. (2018). Heart disease and stroke Statistics-2018 update: a report from the American Heart Association. Circulation.

[3] Vahanian A., Beyersdorf F., Praz F. (2022). 2021 ESC/EACTS guidelines for the management of valvular heart disease. Eur Heart J.

[4] Otto C.M., Nishimura R.A., Bonow R.O. (2021). 2020 ACC/AHA guideline for the management of patients with valvular heart disease: a report of the American College of Cardiology/American Heart Association Joint Committee on clinical practice guidelines. J Am Coll Cardiol.

[5] Coisne A., Lancellotti P., Habib G. (2023). ACC/AHA and ESC/EACTS guidelines for the management of valvular heart diseases: JACC guideline comparison. J Am Coll Cardiol.

[6] Ajmone M.N., Delgado V., Shah D.J. (2023). Valvular heart disease: shifting the focus to the myocardium. Eur Heart J.

[7] Dahl J.S., Christensen N.L., Videbæk L. (2014). Left ventricular diastolic function is associated with symptom status in severe aortic valve stenosis. Circ Cardiovasc Imaging.

[8] Généreux P., Pibarot P., Redfors B. (2017). Staging classification of aortic stenosis based on the extent of cardiac damage. Eur Heart J.

[9] Généreux P., Pibarot P., Redfors B. (2022). Evolution and prognostic impact of cardiac damage after aortic valve replacement. J Am Coll Cardiol.

[10] Généreux P., Cohen D.J., Pibarot P. (2023). Cardiac damage and quality of life after aortic valve replacement in the PARTNER trials. J Am Coll Cardiol.

[11] Tastet L., Tribouilloy C., Maréchaux S. (2019). Staging cardiac damage in patients with asymptomatic aortic valve stenosis. J Am Coll Cardiol.

[12] Amanullah M.R., Pio S.M., Ng A.C.T. (2021). Prognostic implications of associated cardiac abnormalities detected on echocardiography in patients with moderate aortic stenosis. JACC Cardiovasc Imaging.

[13] Dahl J.S., Julakanti R., Ali M., Scott C.G., Padang R., Pellikka P.A. (2024). Cardiac damage in early aortic stenosis: is the valve to blame?. JACC Cardiovasc Imaging.

[14] Coisne A., Montaigne D., Aghezzaf S. (2021). Association of mortality with aortic stenosis severity in outpatients: results from the VALVENOR study. JAMA Cardiol.

[15] Vahanian A., Beyersdorf F., Praz F. (2021). 2021 ESC/EACTS guidelines for the management of valvular heart disease. Eur Heart J.

[16] Coisne A., Montaigne D., Ninni S. (2022). Diabetes mellitus and cardiovascular mortality across the spectrum of aortic stenosis. Heart Br Card Soc.

[17] Coisne A., Montaigne D., Aghezzaf S. (2024). Clinical outcomes according to aortic stenosis management: insights from real-world practice. J Am Heart Assoc.

[18] Bauters C., Tricot O., Meurice T., Lamblin N. (2017). Long-term risk and predictors of cardiovascular death in stable coronary artery disease: the CORONOR study. Coron Artery Dis.

[19] Snir A.D., Ng M.K., Strange G. (2021). Cardiac damage staging classification predicts prognosis in all the major subtypes of severe aortic stenosis: insights from the National echo database Australia. J Am Soc Echocardiogr.

[20] Angellotti D., Ryffel C., Schmid L. (2025). Long-term outcomes in moderate and severe aortic stenosis according to extent of cardiac damage. JACC Cardiovasc Interv.

[21] Tomaselli M., Springhetti P., Benfari G. (2025). A novel staging system of cardiac damage in aortic stenosis based on multi-chamber myocardial deformation. Eur Heart J Cardiovasc Imaging.

[22] Vollema E.M., Amanullah M.R., Ng A.C.T. (2019). Staging cardiac damage in patients with symptomatic aortic valve stenosis. J Am Coll Cardiol.

[23] Abdelfattah O.M., Jacquemyn X., Sá M.P. (2024). Cardiac damage staging predicts outcomes in aortic valve stenosis after aortic valve replacement: meta-analysis. JACC Adv.

[24] Généreux P., Redfors B., Pibarot P. (2025). Health care cost and resource utilization after aortic valve replacement according to the extent of cardiac damage. Circ Cardiovasc Interv.

[25] Myagmardorj R., Fortuni F., Généreux P. (2025). The reversibility of cardiac damage after transcatheter aortic valve implantation and short-term outcomes in a real-world setting. Eur Heart J Cardiovasc Imaging.

[26] Jacob S., Hecht S., Tastet L. (2025). Evolution of cardiac damage following SAVR in asymptomatic severe aortic stenosis. Circ Cardiovasc Interv.

[27] Gillam L.D., Généreux P. (2025). Staging aortic stenosis based on cardiac damage: a new tool for risk prediction, clinical decision-making, and trial design. Struct Heart J Heart Team.

[28] Coisne A., Scotti A., Latib A., Leon M.B., Granada J.F. (2022). Reply: significant vs severe aortic stenosis: the rule of 4. JACC Cardiovasc Interv.

[29] Otto C.M., Burwash I.G., Legget M.E. (1997). Prospective study of asymptomatic valvular aortic stenosis. Clinical, echocardiographic, and exercise predictors of outcome. Circulation.

