# Supplementary material for: Association Between the Degree of Extravalvular Cardiac Damage and the Severity of Aortic Stenosis
Source: JACC Adv. 2026 Sep 10;5(10):103227. doi: 10.1016/j.jacadv.2026.103227 (PMC13586839; doi:10.1016/j.jacadv.2026.103227)
Supplement: Supplementary file 1 [file mmc1.docx]

**Supplemental Material**

**Supplemental Table 1. Comparison of baseline characteristics in patients with staging available vs patients without staging………………….. 3**

**Supplemental Table 2. AVR rates according to extravalvular CD stage (n = 621)** **…………………………………………………………………...4**

**Supplemental Table 3. Baseline characteristics according to cardiac damage staging in patients with mild AS…………………………………...5**

**Supplemental Table 4. Baseline characteristics according to cardiac damage staging in patients with moderate AS……………………………...6**

**Supplemental Table 5. Baseline characteristics according to cardiac damage staging in patients with severe AS…………………….…………...7**

**Supplemental Table 6. Factors Associated With CD Stage >0 With Aortic Stenosis Severity Categories Included in the Model…….…………...8**

**Supplemental Table 7. Cox analysis using CD stage as separate categories, with stage 0 as the reference.…………………….…………..………....9**

**Supplemental Table 8. Analysis on the prognostic impact of CD stage >0 versus CD stage 0 over the entire follow-up period without censoring at the time of AVR (n = 1,747 patients)…………………………………………………………………………………………………….…………...10**

**Supplemental Table 9. Analysis on the prognostic impact of CD stage >0 versus CD stage 0 over the entire follow-up period without censoring at the time of AVR, with adjustment for AVR as a time-dependent covariate (n = 1,747 patients).…………………….…………………………....11**

**Supplemental Table 10. Summary of the extravalvular CD stages in AS patients ………………………………………………………..………...12**

**Supplemental Table 1. Comparison of baseline characteristics in patients with staging available vs patients without staging**

|  | **No staging**  **(n = 957)** | **Staging**  **(n = 1747)** | ***P* value** |
| --- | --- | --- | --- |
| Age (years) | 76.1 ± 10.2 | 76 ± 11.2 | 0.717 |
| Women | 428 (44.7) | 829 (47.5) | 0.174 |
| History of hypertension | 738 (77.1) | 1328 (76) | 0.519 |
| Diabetes mellitus | 301 (31.5) | 519 (29.7) | 0.345 |
| Previous coronary event | 163 (17) | 331 (19) | 0.218 |
| Previous myocardial infarction | 79 (8.3) | 173 (9.9) | 0.159 |
| Previous coronary bypass | 40 (4.2) | 86 (4.9) | 0.381 |
| Previous PCI | 122 (12.8) | 229 (13.1) | 0.790 |
| Previous hospitalization for HF | 70 (7.3) | 199 (11.4) | 0.001 |
| Previous stroke | 80 (8.4) | 150 (8.6) | 0.840 |
| NYHA class at inclusion ^a^:  - 1  - 2  - 3-4 | 402 (42.3)  442 (46.5)  106 (11.2) | 659 (38)  850 (49.1)  224 (12.9) | 0.073 |
| Angina at inclusion | 32 (3.3) | 77 (4.4) |  |
| Vmax (m/s) | 3.27 ± 0.64 | 3.28 ± 0.66 | 0.729 |
| AS severity:  - mild  - moderate  - severe | 412 (43.1)  407 (42.5)  138 (14.4) | 744 (42.6)  714 (40.9)  289 (16.5) | 0.332 |

^a^ missing data in 21 patients

**Supplemental Table 2. AVR rates according to extravalvular CD stage (n = 621)**

|  | **Stage 0**  **(n = 170)** | **Stage 1**  **(n = 123)** | **Stage 2**  **(n = 280)** | **Stage 3**  **(n = 36)** | **Stage 4**  **(n = 12)** | ***P* value** |
| --- | --- | --- | --- | --- | --- | --- |
|  |  |  |  |  |  |  |
| SAVR | 94 (55.3) | 70 (56.9) | 116 (41.4) | 10 (27.8) | 5 (41.7) | 0.001 |
| TAVR | 76 (44.7) | 53 (43.1) | 164 (58.6) | 26 (72.2) | 7 (58.3) |  |

**Supplemental Table 3. Baseline characteristics according to cardiac damage staging in patients with mild AS**

|  | **Stage 0**  **(n = 283)** | **Stage 1**  **(n = 89)** | **Stage 2**  **(n = 305)** | **Stage 3**  **(n = 43)** | **Stage 4**  **(n = 24)** | ***P* value** |
| --- | --- | --- | --- | --- | --- | --- |
| Age (years) | 72.1 ± 12.3 | 76 ± 10 | 77.5 ± 9.9 | 78.8 ± 11.6 | 76.2 ± 10.2 | <0.001 |
| Women | 125 (44.2) | 43 (48.3) | 142 (46.6) | 28 (65.1) | 8 (33.3) | 0.078 |
| History of hypertension | 212 (74.9) | 67 (75.3) | 268 (87.9) | 33 (76.7) | 19 (79.2) | 0.001 |
| Diabetes mellitus | 88 (31.1) | 33 (37.1) | 99 (32.5) | 13 (30.2) | 10 (41.7) | 0.715 |
| Previous coronary event | 45 (15.9) | 25 (28.1) | 70 (23) | 8 (18.6) | 10 (41.7) | 0.007 |
| Previous myocardial infarction | 24 (8.5) | 11 (12.4) | 37 (12.1) | 6 (14) | 6 (25) | 0.118 |
| Previous coronary bypass | 6 (2.1) | 6 (6.7) | 14 (4.6) | 4 (9.3) | 4 (16.7) | 0.004 |
| Previous PCI | 35 (12.4) | 21 (23.6) | 53 (17.4) | 4 (9.3) | 4 (16.7) | 0.073 |
| Previous hospitalization for HF | 8 (2.8) | 8 (9) | 46 (15.1) | 9 (20.9) | 9 (37.5) | <0.001 |
| Previous stroke | 17 (6) | 10 (11.2) | 33 (10.8) | 1 (2.3) | 4 (16.7) | 0.058 |
| NYHA class at inclusion ^a^: |  |  |  |  |  |  |
| - 1 | 148 (52.5) | 40 (45) | 127 (41.8) | 6 (14) | 2 (8.7) |  |
| - 2 | 117 (41.5) | 42 (47) | 150 (49.3) | 23 (53.5) | 8 (34.8) | <0.001 |
| - 3-4 | 17 (6) | 7 (8) | 27 (8.9) | 14 (32.5) | 13 (56.5) |  |
| Angina at inclusion | 7 (2.5) | 3 (3.4) | 8 (2.6) | 2 (4.7) | 0 | 0.825 |
| ß-blocker | 117 (41.5) | 45 (50.6) | 177 (58) | 27 (62.8) | 15 (62.5) | <0.001 |
| ACE-I or ARB | 189 (66.8) | 60 (67.4) | 223 (73.1) | 32 (74.4) | 17 (70.8) | 0.479 |
| Statin | 163 (57.6) | 51 (57.3) | 197 (64.6) | 24 (55.8) | 12 (50) | 0.293 |
| Antiplatelet | 136 (48.1) | 51 (57.3) | 127 (41.6) | 13 (30.2) | 11 (45.8) | 0.020 |
| Oral anticoagulant | 40 (14.1) | 12 (13.5) | 114 (37.4) | 15 (34.9) | 13 (54.2) | <0.001 |

Data are mean ± SD or n (%). ^a^ missing data in 3 patients. ACE-I, angiotensin-converting enzyme inhibitor; ARB, angiotensin 2 receptor antagonist; HF, heart failure; NYHA, New York Heart Association; PCI, percutaneous coronary intervention. P value by ANOVA or Chi-2.

**Supplemental Table 4. Baseline characteristics according to cardiac damage staging in patients with moderate AS**

|  | **Stage 0**  **(n = 190)** | **Stage 1**  **(n = 137)** | **Stage 2**  **(n = 319)** | **Stage 3**  **(n = 47)** | **Stage 4**  **(n = 21)** | ***P* value** |
| --- | --- | --- | --- | --- | --- | --- |
| Age (years) | 73.9 ± 13 | 76.7 ± 9.9 | 78.1 ± 9.6 | 78.7 ± 9.7 | 77.5 ± 9.2 | <0.001 |
| Women | 85 (44.7) | 60 (43.8) | 148 (46.4) | 27 (57.5) | 12 (57.1) | 0.425 |
| History of hypertension | 125 (65.8) | 113 (82.4) | 243 (76.2) | 28 (59.6) | 15 (71.4) | 0.002 |
| Diabetes mellitus | 49 (25.8) | 35 (25.6) | 99 (31) | 5 (10.6) | 10 (47.6) | 0.009 |
| Previous coronary event | 27 (14.2) | 22 (16.1) | 62 (19.4) | 9 (19.2) | 9 (42.9) | 0.022 |
| Previous myocardial infarction | 11 (5.8) | 13 (9.5) | 35 (11) | 5 (10.6) | 6 (28.6) | 0.015 |
| Previous coronary bypass | 5 (2.6) | 7 (5.1) | 18 (5.6) | 4 (8.5) | 5 (23.8) | 0.002 |
| Previous PCI | 20 (10.5) | 12 (8.8) | 41 (12.9) | 7 (14.9) | 3 (14.3) | 0.659 |
| Previous hospitalization for HF | 6 (3.2) | 15 (11) | 37 (11.6) | 11 (23.4) | 10 (47.6) | <0.001 |
| Previous stroke | 15 (7.9) | 5 (3.7) | 42 (13.2) | 1 (2.1) | 1 (4.7) | 0.005 |
| NYHA class at inclusion ^a^: |  |  |  |  |  |  |
| - 1 | 86 (45.3) | 42 (30.7) | 99 (31.3) | 16 (34) | 3 (15) |  |
| - 2 | 85 (44.7) | 78 (56.9) | 176 (55.7) | 23 (49) | 9 (45) | 0.001 |
| - 3-4 | 19 (10) | 17 (12.4) | 41 (13) | 8 (17) | 8 (40) |  |
| Angina at inclusion | 8 (4.2) | 8 (5.8) | 17 (5.3) | 2 (4.3) | 1 (4.8) | 0.966 |
| ß-blocker | 61 (32.1) | 63 (46) | 164 (51.4) | 24 (51.1) | 12 (57.1) | 0.001 |
| ACE-I or ARB | 105 (55.3) | 91 (66.4) | 217 (68) | 32 (68) | 14 (66.7) | 0.054 |
| Statin | 89 (46.8) | 68 (49.6) | 184 (57.7) | 23 (49) | 12 (57.1) | 0.150 |
| Antiplatelet | 90 (47.4) | 71 (51.8) | 153 (48) | 20 (42.6) | 7 (33.3) | 0.527 |
| Oral anticoagulant | 19 (10) | 21 (15.3) | 90 (28.2) | 13 (27.7) | 11 (52.4) | <0.001 |

Data are mean ± SD or n (%). ^a^ missing data in 4 patients. ACE-I, angiotensin-converting enzyme inhibitor; ARB, angiotensin 2 receptor antagonist; HF, heart failure; NYHA, New York Heart Association; PCI, percutaneous coronary intervention. P value by ANOVA or Chi-2.

**Supplemental Table 5. Baseline characteristics according to cardiac damage staging in patients with severe AS**

|  | **Stage 0**  **(n = 51)** | **Stage 1**  **(n = 72)** | **Stage 2**  **(n = 141)** | **Stage 3**  **(n = 19)** | **Stage 4**  **(n = 6)** | ***P* value** |
| --- | --- | --- | --- | --- | --- | --- |
| Age (years) | 75.6 ± 10.5 | 74.9 ± 11.6 | 75.9 ± 12.6 | 81.9 ± 8.8 | 69.7 ± 9.7 | 0.127 |
| Women | 29 (56.9) | 35 (48.6) | 74 (52.5) | 10 (52.6) | 3 (50) | 0.934 |
| History of hypertension | 38 (74.5) | 46 (63.9) | 103 (73.1) | 13 (68.4) | 5 (83.3) | 0.581 |
| Diabetes mellitus | 12 (23.5) | 22 (30.6) | 39 (27.7) | 3 (15.8) | 2 (33.3) | 0.710 |
| Previous coronary event | 6 (11.8) | 11 (15.3) | 18 (12.8) | 5 (26.3) | 4 (66.7) | 0.004 |
| Previous myocardial infarction | 4 (7.8) | 5 (6.9) | 5 (3.6) | 2 (10.5) | 3 (50) | <0.001 |
| Previous coronary bypass | 2 (3.9) | 2 (2.8) | 5 (3.6) | 1 (5.3) | 3 (50) | <0.001 |
| Previous PCI | 3 (5.9) | 9 (12.5) | 13 (9.2) | 3 (15.8) | 1 (16.7) | 0.635 |
| Previous hospitalization for HF | 4 (7.8) | 7 (9.7) | 21 (14.9) | 6 (31.6) | 2 (33.3) | 0.047 |
| Previous stroke | 4 (7.8) | 2 (2.8) | 12 (8.5) | 2 (10.5) | 1 (16.7) | 0.465 |
| NYHA class at inclusion ^a^: |  |  |  |  |  |  |
| - 1 | 18 (36) | 22 (31.4) | 46 (33.3) | 3 (16.7) | 1 (16.7) |  |
| - 2 | 25 (50) | 37 (52.9) | 65 (47.1) | 8 (44.4) | 4 (66.6) | 0.457 |
| - 3-4 | 7 (14) | 11 (15.7) | 27 (19.6) | 7 (38.9) | 1 (16.7) |  |
| Angina at inclusion | 1 (2) | 8 (11.1) | 10 (7.1) | 2 (10.5) | 0 | 0.344 |
| ß-blocker | 14 (27.5) | 21 (29.2) | 57 (40.4) | 7 (36.8) | 5 (83.3) | 0.038 |
| ACE-I or ARB | 31 (60.8) | 42 (58.3) | 83 (58.9) | 11 (57.9) | 5 (83.3) | 0.821 |
| Statin | 24 (47.1) | 32 (44.4) | 67 (47.5) | 12 (63.2) | 5 (83.3) | 0.275 |
| Antiplatelet | 28 (54.9) | 31 (43.1) | 58 (41.1) | 6 (31.6) | 3 (50) | 0.377 |
| Oral anticoagulant | 6 (11.8) | 11 (15.3) | 39 (27.7) | 7 (36.8) | 2 (33.3) | 0.035 |

Data are mean ± SD or n (%). ^a^ missing data in 7 patients. ACE-I, angiotensin-converting enzyme inhibitor; ARB, angiotensin 2 receptor antagonist; HF, heart failure; NYHA, New York Heart Association; PCI, percutaneous coronary intervention. P value by ANOVA or Chi-2.

**Supplemental Table 6. Factors Associated With CD Stage >0 With Aortic Stenosis Severity Categories Included in the Model**

|  | **Univariable** | | **Multivariable** | |
| --- | --- | --- | --- | --- |
|  | **OR (95% CI)** | ***P* value** | **OR (95% CI)** | ***P* value** |
| Age, per year | 1.03 (1.02-1.04) | <0.001 | 1.02 (1.01-1.03) | <0.001 |
| Women | 1.11 (0.91-1.36) | 0.313 | 0.96 (0.76-1.21) | 0.710 |
| History of hypertension | 1.40 (1.11-1.77) | 0.004 | 1.30 (1.00-1.67) | 0.049 |
| Diabetes mellitus | 1.09 (0.87-1.37) | 0.446 | 1.06 (0.83-1.35) | 0.637 |
| Previous coronary event | 1.49 (1.13-1.97) | 0.005 | 1.34 (0.99-1.81) | 0.056 |
| Previous hospitalization for HF | 4.88 (2.97-8.02) | <0.001 | 3.98 (2.39-6.61) | <0.001 |
| Previous stroke | 1.39 (0.94-2.06) | 0.095 | 1.19 (0.79-1.79) | 0.405 |
| NYHA class >1 | 1.84 (1.50-2.28) | <0.001 | 1.29 (1.03-1.62) | 0.028 |
| Angina at inclusion | 1.67 (0.95-2.92) | 0.074 | 1.27 (0.71-2.29) | 0.420 |
| Mild AS | reference | - | reference | - |
| Moderate AS | 1.69 [1.36 - 2.11] | <0.001 | 1.67 (1.32-2.11) | <0.001 |
| Severe AS | 2.86 [2.05 - 4.01] | <0.001 | 2.92 (2.06-4.16) | <0.001 |

HF, heart failure; NYHA, New York Heart Association

**Supplemental Table 7. Cox analysis using CD stage as separate categories, with stage 0 as the reference (with censoring at the time of AVR)**

|  | **HR (95% CI)** | ***P*** |  |
| --- | --- | --- | --- |
| **All-cause mortality** |  |  |  |
| CD stage:  0  1  2  3  4 | reference  1.34 (1.02-1.76)  1.65 (1.34-2.02)  2.77 (2.02-3.80)  4.87 (3.35-7.06) | -  0.034  <0.001  <0.001  <0.001 |  |
| **Cardiovascular mortality** |  |  |  |
| CD stage:  0  1  2  3  4 | reference  2.23 (1.44-3.45)  2.21 (1.54-3.18)  4.13 (2.49-6.86)  7.64 (4.32-13.5) | -  <0.001  <0.001  <0.001  <0.001 |  |
|  |  |  |  |

**Supplemental Table 8. Analysis on the prognostic impact of CD stage >0 versus CD stage 0 over the entire follow-up period without censoring at the time of AVR (n = 1,747 patients)**

|  | **HR (95% CI)** | ***P*** |  |
| --- | --- | --- | --- |
| All-cause mortality | 1.68 [1.41 - 2.00] | <0.001 |  |
| Cardiovascular mortality | 2.45 [1.80 - 3.33] | <0.001 |  |
|  |  |  |  |

**Supplemental Table 9. Analysis on the prognostic impact of CD stage >0 versus CD stage 0 over the entire follow-up period without censoring at the time of AVR, with adjustment for AVR as a time-dependent covariate (n = 1,747 patients).**

|  | **HR (95% CI)** | ***P*** |  |
| --- | --- | --- | --- |
| **All-cause mortality** |  |  |  |
| CD stage >0 | 1.76 [1.47 - 2.10] | <0.001 |  |
| AVR | 0.56 [0.46 - 0.69] | <0.001 |  |
| **Cardiovascular mortality** |  |  |  |
| CD stage >0 | 2.49 [1.83 - 3.38] | <0.001 |  |
| AVR | 0.84 [0.63 - 1.11] | 0.221 |  |
| AVR was modelled as a time-dependent covariate |  |  |  |

**Supplemental Table 10. Summary of the extravalvular CD stages in AS patients**

| **First Author** | **PMID** | **Year** | **Number of patients** | **AS severity** | **Stage 0**  **(%)** | **Stage 1**  **(%)** | **Stage 2**  **(%)** | **Stage 3**  **(%)** | **Stage 4**  **(%)** |
| --- | --- | --- | --- | --- | --- | --- | --- | --- | --- |
| Généreux P | 29020232 | 2017 | 1661 | severe | 2.8 | 12.8 | 50.8 | 24.9 | 8.7 |
| Tastet L | 31345430 | 2019 | 735 | Moderate, severe | 12 | 27 | 46 | 14 (stage 3 et 4) | |
| Vollema EM | 31345429 | 2019 | 1189 | severe | 8 | 24 | 49 | 7 | 12 |
| Fukui M | 30725109 | 2019 | 689 | severe | 0 | 13 | 62 | 21 | 4 |
| Berkovitch A | 32008849 | 2020 | 2608 | severe | 29 | 30 | 28 | 12.3 | 1.2 |
| Maeder MT | 32416065 | 2020 | 429 | severe | 16 | 27 | 36 | 17 | 4 |
| Amanullah MR | 34023268 | 2021 | 1245 | moderate | 13.1 | 26.8 | 42.6 | 10.6 | 6.9 |
| Schewel J | 33744987 | 2021 | 1400 | severe | 9.9 | 23.6 | 33.5 | 24.1 | 10 |
| Okuno T | 34086888 | 2021 | 1133 | severe | 1.2 | 3.3 | 9.1 | 24 | 62.4 |
| Dahl J | 38904570 | 2024 | 9611 | mild | 20 | 17 | 50 | 9 | 4 |
| Angellotti D | 41161924 | 2025 | 343  283 | moderate  severe | 7.9  6.4 | 14.3  12.4 | 51.6  48.8 | 10.8  15.9 | 15.5  16.6 |
| Valvenor |  | 2025 | 1747 | mild, moderate, severe | 30 | 17 | 44 | 6 | 3 |
